# Syphilitic orchitis mimicking a testicular tumor in a clinically occult HIV-infected young man: a case report with emphasis on a challenging pathological diagnosis

**DOI:** 10.1186/s13000-016-0454-x

**Published:** 2016-01-14

**Authors:** Chia-Ying Chu, Wei-Yu Chen, Shauh-Der Yeh, Huey-Min Yeh, Chia-Lang Fang

**Affiliations:** Department of Pathology, Taipei Medical University Hospital, Taipei, Taiwan; Department of Pathology, School of Medicine, College of Medicine, Taipei Medical University, Taipei, Taiwan; Department of Pathology, Wan Fang Hospital, Taipei Medical University, Taipei, Taiwan; Department of Urology, Taipei Medical University Hospital, Taipei, Taiwan; Department of Radiology, Taipei Medical University Hospital, 250 Wu Hsing Street, Taipei, 110 Taiwan

**Keywords:** Syphilitic orchitis, Gumma, Interstitial orchitis, HIV infection

## Abstract

**Background:**

Syphilitic orchitis is a rare manifestation of gumma in tertiary syphilis, microscopically typically characterized by multiple discrete granulomas with central necrosis and peripheral fibrosis. We report a case of syphilitic orchitis mimicking a testicular tumor with atypical histological features.

**Case presentation:**

A 33-year-old clinically occult HIV-infected man had a testicular tumor. A radical orchiectomy was performed, and a histological examination showed an acute and chronic interstitial inflammatory lesion as well as spindle cell proliferation, without typical gumma formation, necessitating the differential diagnosis having to be made from a panel of etiological factors. Syphilitic orchitis was confirmed by both an immunohistochemical study and PCR testing for the Treponema pallidum DNA polymerase I gene using paraffin-embedded tissues. However, serology tests, including both the Venereal Disease Research Laboratory (VDRL) test and Treponema pallidum partical agglutination (TTPA), demonstrated false-negative results.

**Conclusion:**

Syphilitic orchitis may present atypical and unusual histological features, and should be included in the differential diagnoses of nonspecific interstitial inflammatory lesions of the testes by pathologists, especially in immunocompromised patients.

## Background

Syphilitic orchitis is an uncommon but well-known etiology of infectious granulomatous orchitis. Two different morphological patterns are recognized in syphilitic orchitis: (1) a fibrotic type and (2) a gummatous type. The fibrotic type is characterized by interstitial peritubular lymphoplasma cell infiltration and peritubular fibrosis. In the gummatous type, discrete gummas can be seen grossly. Microscopically, the gummas are composed of a central zone of coagulative necrosis surrounded by lymphoplasma cells, histiocytes, and occasional epithelioid giant cells with peripheral fibrosis. Obliterative endarteritis may also be present [1]. Herein, we report a case of syphilitic orchitis clinically presenting as a testicular tumor with atypical and unusual microscopic features pathologically simulating other entities in a young occult HIV-infected man.

## Case presentation

### Clinical summary

**Fig. 1:**
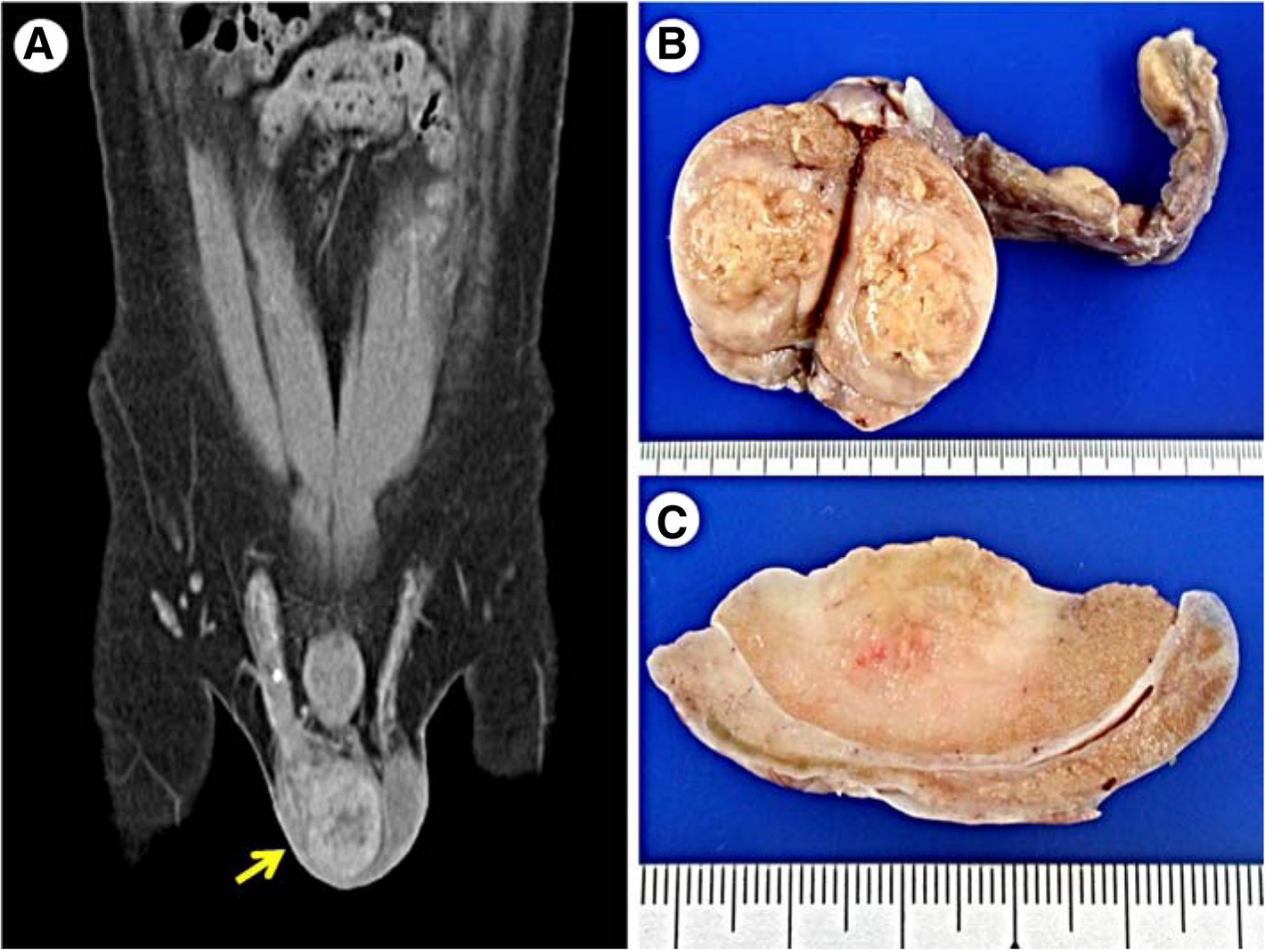
**a** Contrast CT shows enlargement of right testis with heterogeneous enhancement (*arrow*). **b** The testis shows a gray and yellow bulging tumor on cut surface. **c** The border of the tumor is infiltrative

A 33-year-old man presented with right scrotal hardening and swelling, accompanied by inguinal pain and flank soreness, for 3 months. An off and on fever was also noted. Right epididymitis was the initial impression, and oral antibiotics of Cephalexin were prescribed. The symptoms persisted after treatment. An ultrasound examination showed heterogeneous echogenicity of the right testis, suggesting a testicular tumor. A further pelvic computed tomographic scan revealed enlargement of the right testis estimated to be 4.5 x 4.0 cm in dimension, with heterogeneous enhancement, suspected of being a testicular tumor (Fig. 1a). No abdominal lymphadenopathy was found. Serum tumor markers, including α-fetoprotein, β-human chorionic gonadotropin (β-hCG) and lactate dehydrogenase (LDH), were within normal limits. A right radical orchiectomy was performed under the impression of a testicular tumor.

### Gross and microscopic findings

**Fig. 2:**
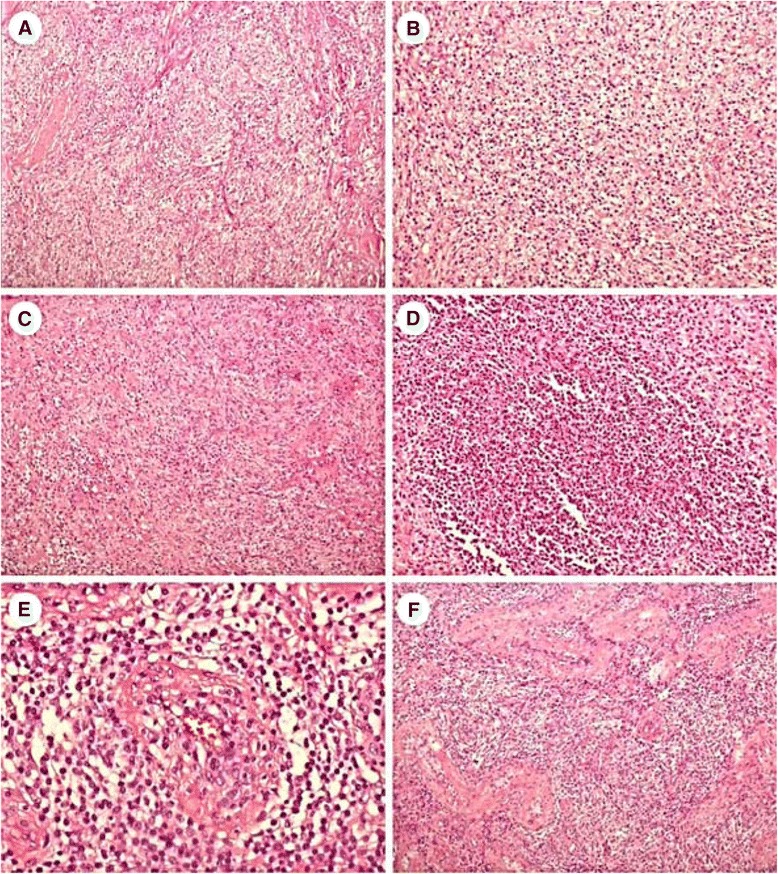
**a** At low power, the testicular tumor shows an inflammatory lesion. **b** Higher magnification shows lymphoplasma cell and histiocyte infiltration. **c** Spindle cell proliferation in short fascicles is seen in areas. **d** Microabscess formation is focally evidenced. **e** Obliterative vasculitis is occasionally found. **f** In the periphery of the tumor, interstitial inflammatory cell infiltration and tubular atrophy are present (original magnification, H&E × 100 [**a**, **c** and **f**], ×200 [**b** and **d**], ×400 [**e**])

On gross examination, the testis revealed an ill-defined, gray to tan, soft-tumor-like lesion measuring 3.3 x 3.2 x 2.6 cm (Fig. 1b, c). Histologically, the lesion disclosed dense lymphoplasma cell and histiocyte infiltration in the interstitium, as well as spindle cell proliferation (Fig. 2a, b, c). There were small foci of foamy histiocyte aggregation and microabscess formation in the lesion (Fig. 2d). Some of the blood vessels revealed obliterative vasculitis (Fig. 2e). The seminiferous tubules revealed atrophic change (Fig. 2f). There was no well-formed granuloma or caseous necrosis in the lesion. The inflammatory process had also partially affected the epididymis and spermatic cord.

### Immunohistochemistry

**Fig. 3:**
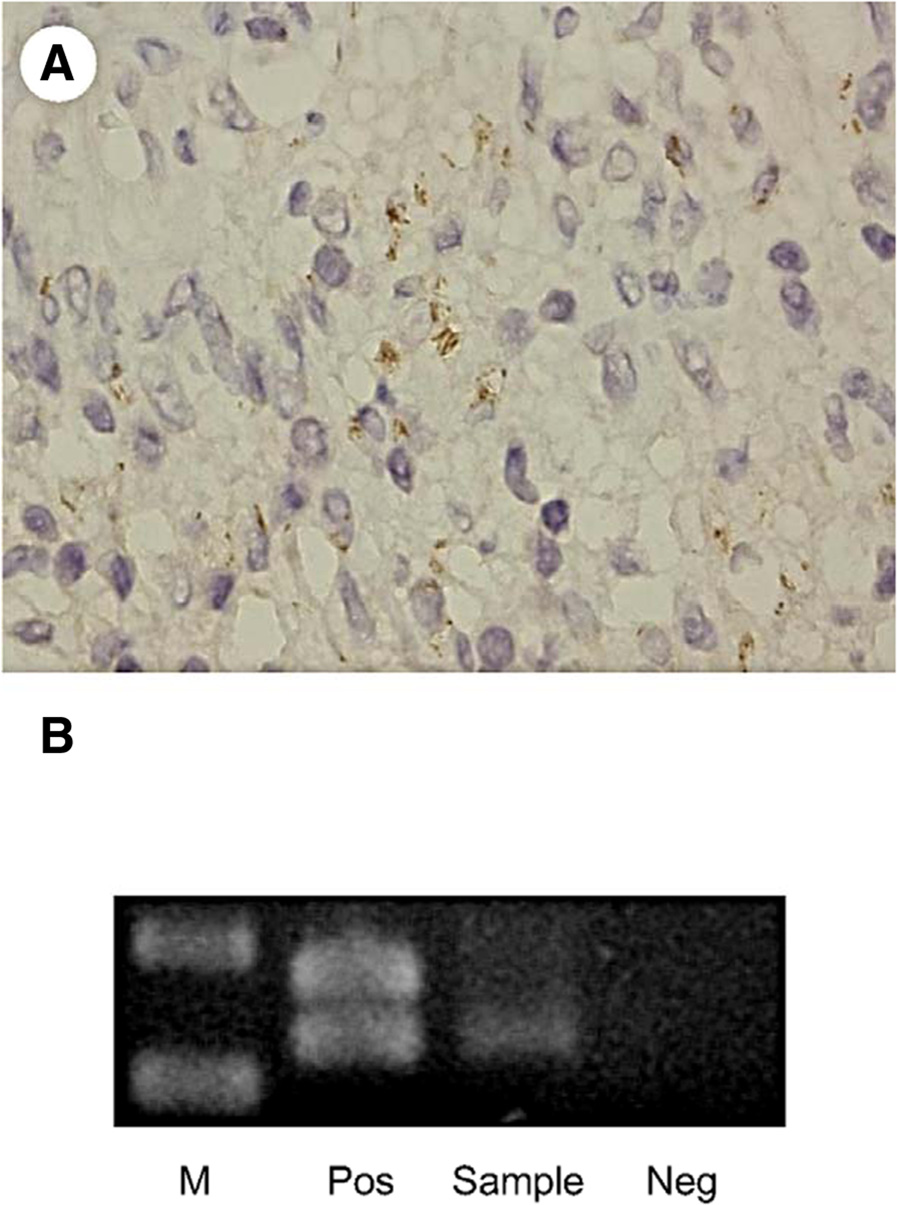
**a** Immunostain of syphilis shows spirochetes in the cytoplasm of histiocytes (1000 X, oil immersion). **b** DNA expression of TPmod1 is detected by PCR analysis. (Pos: positive control; Sample: tumor of the patient; Neg: negative control)

A panel of histochemical stains and immunohistochemical (IHC) stains was performed. IHC staining of CD68 (1:250; Biocare) and CD163 (1:50; Leica) proved a histiocytic nature. The spindle cells were focally positive for smooth muscle actin (1:50; DAKO), but negative for ALK (1:200; NOVO) and CD21 (1:100; Cell Marque), suggesting a myofibroblastic origin. No significant immunoglobulin G4 -positive plasma cell infiltration was seen (IgG4, 1:800; Invitrogen). No microorganisms were demonstrated by acid-fast, periodic acid-Schiff, Grocott-Gomori methenamine-silver stains, or Gram’s stain. IHC with a rabbit polyclonal antibody against *Treponema pallidum* (dilution 1:100, Biocare Medical, Concord, CA, USA) displayed some spirochetes in the cytoplasm of histiocytes (Fig. 3a), highly suggestive of syphilitic orchitis.

### *Treponema pallidum* DNA polymerase I polymerase chain reaction

Polymerase chain reaction (PCR) testing for the *T. pallidum* DNA polymerase I gene using paraffin-embedded tissue was done. Positive control was applied using an anal biopsy specimen previously diagnosed as syphilis proved by immunohistochemistry and serology. A semi-nested PCR using the primers of TPmod-F1 (5′-GTGTGCACTGGGCATTACAG-3′), TPmod-F2 (5′-TGAAGCTGACGACCTCATTG-3′), and TP mod-R1 (5′- GTCTGAGCACTTGCACCGTA-3′) targeted a different region of the *T. pallidum* DNA polymerase I gene (Fig. 3b) [2]. Direct sequencing proved positivity for *T. pallidum,* which confirmed the diagnosis of syphilitic orchitis.

The patient underwent further serology tests postoperatively. An anti-human immunodeficient virus (HIV) enzyme-linked immunosorbent assay (ELISA) and Western blot tests were positive. However, serology tests for syphilis, including both Venereal Disease Research Laboratory (VDRL) test and *Treponema pallidum* particle agglutination (TPPA), demonstrated false-negative results. At that time, the patient had a low CD4 count (72 cells/mm^3^) and a low CD4/CD8 ratio (0.07).

## Discussion

Syphilitic orchitis is a rare manifestation of gummas in patients with tertiary syphilis. Syphilitic gummas may present as a testicular mass and mimic malignant neoplasms clinically. Until recently, fewer than 20 cases had been reported in the English literature [3].

Microscopic features of syphilitic gummas, which are characterized by granulomatous inflammation with central necrosis and peripheral fibrosis, belong to the spectrum of granulomatous orchitis. In our case, the gross and microscopic findings were atypical for syphilitic gummas, but rather nonspecific interstitial infiltration of lymphoplasma cells, histiocytes, and foamy histiocytes, associated with microabscesses and spindle cell proliferation. A number of etiologies and morphological simulating entities should be considered in the differential diagnosis.

Malakoplakia usually occurs in patients with immunosuppression or a history of a prior urinary tract infection. The histological features are characterized by dense epithelioid histiocyte infiltration in the seminiferous tubules and interstitium. Histiocytes have foamy and eosinophilic granular cytoplasm, termed von Hansemann histiocytes. Some of the histiocytes contain basophilic, laminated, and mineralized concretions in the cytoplasm, so-called Michaelis-Gutmann bodies, which can be highlighted by periodic acid-Schiff, von Kossa, and iron stains [4, 5]. The specialized von Hansemann histiocytes and Michaelis-Gutmann bodies were not found in the current case.

Rosai-Dorfman disease rarely involves the testes, and may be clinically confused with neoplasms. Microscopically, characteristic histiocytes with centrally placed nuclei, small nucleoli, and abundant pale eosinophilic cytoplasm infiltrating in the testicular interstitium are seen. The diagnostic feature is emperipolesis with lymphocytes in the cytoplasm of the histiocytes. Immunohistochemically, the histiocytes are diffusely positive for S-100 and CD68, but negative for CD1a [5, 6]. Although considerable numbers of histiocytes were noted in this case, emperipolesis could not be identified.

IgG4-related sclerosing disease is a fibroinflammatory tumorous lesion involving multiple sites. The histological features include dense lymphoplasmacytic infiltration, storiform fibrosis, and obliterative phlebitis. In an IHC study, there are increased IgG4 positive plasma cells (>10 per 10 high power field). This multiorgan disease may involve the genitourinary tract, but testicular or paratesticular involvement is rare [7]. In this case, no significant IgG4-positive plasma cell infiltration was seen.

In addition to a background of dense mixed inflammatory cell infiltration, spindle cell proliferation was also seen in our case. Thus, a panel of spindle cell tumors in an inflammatory background should be considered in the differential diagnosis. Inflammatory myofibroblastic tumors, rarely seen in the testes, are characterized by proliferation of spindle fibroblastic-myofibroblastic cells in a fascicular pattern, admixed with inflammatory cells, including lymphocytes, plasma cells, and eosinophils, infiltrating in a myxoid or collagenous stroma [8]. Immunohistochemically, the spindle tumor cells show variable staining for smooth muscle actin, desmin, and ALK. In this case, the spindle cell area revealed positivity of smooth muscle actin, suggesting a myofibroblastic nature. ALK positivity occurs in about 50% of cases of inflammatory myofibroblastic tumors [8]. Although ALK immunoreactivity was negative in this case, the possibility of an inflammatory myofibroblastic tumor could not completely be excluded.

Follicular dendritic cell sarcomas are also composed of ovoid to spindle tumor cells with vesicular nuclei, small nucleoli, palely eosinophilic cytoplasm, and indistinct cell border arranged in fascicular and storiform patterns. The background shows prominent admixed lymphocytes [9]. Immunohistochemically, tumor cells express dendritic cell markers, including CD21, CD23, and CD35, which were negative in the spindle cell population in the present case.

Interdigitating dendritic cell sarcomas, exceeding rare tumors that share similar histological features with follicular dendritic cell sarcomas, were reported in the testes [10]. Immunohistochemically, this sarcoma expresses S-100, CD1a, and vimentin, but is consistently negative for follicular dendritic cell markers. In this case, no S-100 or CD1a immunoreactivity was detected in the spindle cells.

## Conclusion

Syphilitic gummas, a granulomatous type of tertiary syphilis, can clinically mimic testicular tumors. The histological differential diagnosis includes granulomatous orchitis of various etiologies. Syphilitic orchitis of the nongranulomatous type, as in the current case, features a nonspecific inflammatory reaction and spindle cell proliferation; the differential diagnosis extends to a spectrum of spindle cell tumors intermingled with dense infiltration of inflammatory cells. In such difficult diagnostic cases, a panel of histochemical and IHC studies, with the aid of clinical information and laboratory tests, can achieve a correct diagnosis. Especially in HIV-positive patients, serum VDRL and RPR tests may show false-negative results, as in this case. PCR testing for the *T. pallidum* DNA polymerase I gene using paraffin-embedded tissue is a sensitive and specific method for diagnosing syphilitic orchitis. From this case, both clinicians and pathologists can learn that syphilitic orchitis should be one of the differential diagnoses in an immunocompromised patient with a testicular tumor, even in the absence of gummatous granulomatous inflammation.

## Consent

Written informed consent was obtained from the patient for publication of this Case Report and all accompanying images. A copy of the written consent is available for review by the Editor-in-Chief of this journal.
